# Strategies to improve pharmaceutical supply chain resilience under politico-economic sanctions: the case of Iran

**DOI:** 10.1186/s40545-021-00341-8

**Published:** 2021-07-05

**Authors:** Peivand Bastani, Zahra Dehghan, Seyyed Mansoor Kashfi, Hesam Dorosti, Mohammadtaghi Mohammadpour, Gholamhossein Mehralian, Ramin Ravangard

**Affiliations:** 1 Health Human Resources Research Center, School of Management and Medical Information Sciences, Shiraz University of Medical Sciences, Shiraz, Iran; 2 MPH, Shiraz University of Medical Sciences, Shiraz, Iran; 3 MPH, Faculty of Health, Shiraz University of Medical Sciences, Shiraz, Iran; 4 Student Research Committee, School of Management and Medical Information Sciences, Shiraz University of Medical Sciences, Shiraz, Iran; 5 School of Pharmacy, Shahid Beheshti University of Medical Sciences, Tehran, Iran

**Keywords:** Pharmaceutical supply chain, Resilience, Politico-economic sanctions, Strategy, Qualitative content analysis, Iran

## Abstract

**Background:**

Given the impact of politico-economic sanctions on the pharmaceutical supply chain, this study aims to identify practical strategies to improve the resilience of the Iranian supply chain in pharmaceutical procurement under politico-economic sanctions.

**Methods:**

This is a qualitative content analysis study conducted in 2018. Semi-structured interviews were conducted using snowball sampling, and saturation was achieved after 18 interviews. Guba and Lincoln's criteria, namely credibility, confirmability, transferability, and dependability, were considered to ensure the validity and transparency of the study. A five-step framework analysis was applied to analyze the data using MAX QDA10.

**Results:**

The results led to the identification of nine main themes and 26 subthemes as strategies to improve the resilience of the pharmaceutical chain. According to the thematic map, some of these strategies have an extra-sectoral character: ‘insurance organizations’, ‘strengthening relations with other countries’, ‘mechanization of the distribution system’, and ‘suppliers and manufacturers’. At the same time, some inter-sectoral strategies can help the pharmaceutical chain maintain its resilience: ‘healthcare management and policy’, ‘exploiting local potential’, ‘pricing’, and ‘integrated health information systems.’ As a strategy, ‘Medical community and consumers’ also plays a crucial role in this regard. According to the subthemes, revisions of health management, more supervision, privatization, clinical policies, strategic purchasing, improvement of the referral system, inter-sectoral cooperation, support of indigenous medicines, rational pricing, insurance system, improvement of medical coverage, and development of electronic prescription should be considered by health systems. Sufficient support for indigenous medication and supervision of the distribution system should be considered by the pharmaceutical industry, taking into account the cooperation between consumers and patients.

**Conclusions:**

Integration of the pharmaceutical supply chain and modern technologies, more attention to business complexity, economic development, intense competition, rapid changes in customer needs, and appropriate relationship between manufacturers, distributors, prescribers, and insurance organizations as purchasers should be considered by policymakers to improve supply chain resilience.

## Background

Resilience as a multidisciplinary concept is the ability of a system, community, or even an individual not only to absorb a shock but to adapt by changing its characteristics and improve its readiness to reduce the negative impact of future shocks and impending risks [1]. Resilience is considered the most important and strategic issue for healthcare systems in general and the pharmaceutical supply chain, in particular, to respond to external shocks [2]. In a scoping review, a comprehensive framework for the resilience of the whole healthcare system was presented. According to this theoretical framework, six main issues can influence the health system's resilience: leadership and governance, information, financing, health workforce, service delivery, and medical products such as drugs and medical devices [3].

Given the emphasis on the role of pharmaceuticals on the resilience of health systems, resilience provides the opportunity for the pharmaceutical supply chain to return to its initial position or even to a more optimal position than in the past. Moreover, it can bring about the ability to identify threats to predict the possible losses or defects and head off their recurrence [4].

Resilience in pharmaceutical supply chains is a formidable challenge in almost all countries [5]. Furthermore, it has been proven that any flaw in the pharmaceutical supply chain can lead to catastrophic consequences such as the death or disability of patients [6]. Against this backdrop, it is necessary to identify and categorize the factors contributing to the vulnerability of the pharmaceutical supply chain. Generally speaking, factors that can lead to supply chain disruption can be categorized into three main dimensions: economic, manufacturing, and legal challenges [7]. At the same time, evidence shows environmental forces can also make additional uncertainty in the management of medicine supply chains [8].

In addition to recognizing the disruptive factors, several strategies have been proposed to improve pharmaceutical supply chain resilience, including company commitment to additional production, maintaining safety stock, and strengthening supply chain breakpoints [9]. Information technology, organizational structures, regulatory affairs, and management commitment are also mentioned as other strategies [10]. Although these strategies are consistently focused on manufacturing companies, they can be applied to maintain the resilience of the pharmaceutical supply chain, too [11].

As is often the case with macroeconomic crises or instability, politico-economic sanctions imposed on some underdeveloped or developing countries would seriously affect the resilience of the pharmaceutical supply chain and make it more vulnerable to internal or external changes [12]. Politico-economic sanctions can also lead to an economic crisis, especially in low- or middle-income countries with poor infrastructure. These destructive effects are more pronounced in the health system and people’s access to medicines and medical devices [13]. Although medicines and medical equipment are purportedly exempt from politico-economic sanctions, patient's access to vital drugs and medical equipment is severely impacted due to transportation barriers, cash transfer challenges, and investment restrictions [14].

Iran is a middle-income country with a population of approximately 80 million that has gone through different phases of crippling and relentless politico-economic sanctions imposed by the United States (US) and European Union (UN) since the Islamic Revolution in 1979, with the situation worsening since November 2018 following the US withdrawal from the Joint Comprehensive Plan of Actions (JCPOA) agreement. This has significantly affected Iran’s access to some essential medicines. For instance, a report showed that from 2012 to 2015, access to 13 essential therapeutic medicines under 26 pharmaceutical items was restricted [15].

Accordingly, policymakers need to pay enough attention to the far-reaching implications of the politico-economic sanctions for the pharmaceutical supply chain. Therefore, this study aims to determine practical strategies to enhance the resilience of the pharmaceutical supply chain, focusing on procurement during politico-economic sanctions.

## Methods

This qualitative content analysis was conducted in 2018 to explore applied strategies to improve pharmaceutical supply chain resilience under politico-economic sanctions against Iran.

For this purpose, semi-structured interviews were conducted with presidents, vice presidents, and heads of Iran's Food and Drug Administration. The interviewees were selected using the purposeful and snowball sampling method. First, we interviewed the vice president of the Vice-Chancellor's Office for Food and Drug at Shiraz University of Medical Sciences and the department managers; then, we asked them to introduce people interested in this field. We then selected the study participants from well-informed individuals who were willing to share their opinions. The inclusion criteria were sufficient information, knowledge, and experience about the storage, distribution, and delivery of medicines and the challenges of the pharmaceutical supply chain.

For data collection, individual interviews were conducted with participants, preferably at their workplaces by arrangement. The interviews were conducted in Persian as this is the formal language and mother tongue of all participants. At the beginning of the interviews, general explanations about the aims of the study and the need for confidentiality of information were given verbally. Written informed consent was also obtained from all interviewees, and it was ensured that they could withdraw from the study at any time. Interviews lasted a minimum of 50 min. All interviews were conducted by one of the researchers. They were recorded with the consent of the participants and transcribed within a very short time after completion. Interviews continued until saturation, which was reached after 18 interviews. The saturation level in qualitative studies is when no new concept can emerge, and the latest data added to the previous ones is simply a duplication. Since data collection and data analysis were simultaneous, after 18 individual interviews, the saturation level of the available data was reached, and the process of data collection was interrupted.

In preparing the semi-structured interview guide, a review of the relevant literature was conducted along with an open-ended pilot interview; it was then confirmed by two experts from the Food and Drug Administration, who are affiliated to Shiraz University of Medical Sciences. In addition, the validity of the interview guide was confirmed after conducting two pilot interviews with participants.

To increase the accuracy and precision of the study, four criteria developed by Guba and Lincoln were considered, including credibility, confirmability, transferability, and reliability [16]. To increase the credibility of the study, long-term participation and continuous observation were used so that the researchers were fully involved in the study, appropriate communication was established with the participants, and general concepts that emerged during the study were accepted. In addition, interviews and reviews of the related literature were integrated to accomplish this purpose. To increase the confirmability of the findings, coded data were presented to the participants to verify their accuracy. The transferability of the study results was confirmed by explaining the conditions of the informed study participants and the interview method in an understandable manner. An attempt was also made to fully select the participants according to the study's objectives and our primary inclusion criteria. The data were analyzed simultaneously in parallel with data collection to help the researchers be fully informed about the principles of theoretical research. To increase the reliability of the study findings, the concepts, themes, and audio and textual information were coded. To ensure this, two members of the research team analyzed the content individually. They discussed the themes to reach a consensus in case of disagreement. When the coding process was complete, all initial codes were discussed by all research team members who had no conflict of interest regarding the topic. Finally, two qualitative experts finalized the codes.

In addition, data analysis was conducted in five steps. First, the researcher listened to the audio files of the interview sessions, and the transcribed texts were read to identify the data. In the second step and to identify a thematic framework, repetitive ideas were reduced into groups of similar ideas or codes in the process of identification. In other words, through a decreasing process, the researchers grouped the whole text into meaningful units that reflected the answer to the research question. Then the initial codes were extracted from those meaningful units. Third, indexing units or portions of the data associated with a particular code were characterized. As mentioned earlier, the initial codes were then discussed by all members of the research team and two qualitative experts to arrive at the final codes. In the fourth step, the data were summarized in a table based on the thematic framework, which included the final codes, subthemes, and main themes. Finally, in the fifth step, the data were combined. Then diagrams and interpretations were used to define the concepts, show the relationships between the concepts, specify the nature of the phenomenon, and offer explanations and suggestions [17]. Coding and classification of data were performed using MAX QDA (version 10) software. All transcribed interview content was translated into English for better use of the data analysis software.

This study was approved by the Shiraz University of Medical Sciences Ethics Committee (Code: IR. SUMS. REC 0.1397.18779). Informed consent was obtained verbally from all participants.

## Results

The results led to identifying nine main themes, 26 subthemes, and several codes related to coping strategies to increase resilience in the pharmaceutical supply chain, as shown in Table 1.

**Table 1:** Strategies to increase the pharmaceutical supply chain resilience to sanctions

Main themes	Subthemes	Final codes
Health policy and management	Revisions in Iran’s health management	Ban the importation of drugs that can be produced domestically
		Elimination of the pharmaceutical mafia in the Ministry of Health
		Consideration of health economics
		Planning for funding and transportation of funds
		Improving the resilience of the pharmaceutical system and preparing appropriate plans
		More attention to the efficiency of the system
		Reducing the authorization to establish pharmacies and clinics to prevent induced demand
	Increasing and strengthening oversight	Incorporating information and monitoring systems into a health problem
		Increasing the efficiency of control instruments for pharmacies and clinics
		Harmonization of control instruments in medical universities
		Rapid detection of corruption and precise and targeted fight against it
		Prevention of prescriptions against the pharmacopeia
		Control with the aim of improvement and not punishment
	Provision of clinical guidelines	Considering clinical guidelines as a lost chain in Iran
		Considering clinical guidelines as a tool for treatment management
		Customizing consideration of clinical guidelines according to local needs
	Delegation and privatization	Delegation to medical universities by the Ministry of Health
		Strengthening the authority of the private sector and pharmacists
		Decentralization
		Outsourcing of hospital pharmacies
		Privatization of services
	Centralized and strategic purchasing	Use of centralized and pooled procurement to get great deals
		Need for information transparency with pooled and centralized purchasing
		Separation of purchaser and observer
		Private or semi-public holdings
		Separation of pharmaceutical accounts from other accounts
		Separate payment for different entities
	Proper implementation of the GP plan and referral system	Existing government for patient health management
		Correction of the primary care physician and referral system
		Prevention of patient perplexity in choosing doctor and medications
		Existing systematic relationship between the referral system and the food and drug authority
Utilization of potentials and infrastructure in Iran	Strengthening of inter-sectoral cooperation	National tender for the production of medical devices
		Use of military facilities to manufacture medical devices
		Creation of relationships between the medical industry, the Department of Defense, the electronics industry, and universities
		Allocating contracts to universities to manufacture pharmaceuticals according to their potentials
	More attention to mass media	The crucial role of the media in improving the situation under sanctions and bringing peace to the society
		The positive role of the media in society justification and decreasing crises
		Improving public relations in medical universities in the field of pharmaceuticals
		Creating a free space for information flow and preventing political favoritism
		The crucial role of the media in increasing politicians’ accountability
		Accurate news reporting to increase public confidence
		Use of competent advisors to health managers in the media field
	Relying on national and religious power and authority	Improving religious and patriotic passions and enthusiasm
		Improvement of sympathy and empathy in society
		Use of social and human capital
		Improving people's self-assessment
Relations with other countries	Interactions with neighboring countries	Planning to take advantage of neighbors
		Increasing cooperation with Islamic countries and the Middle East
		Increasing cooperation with Islamic countries and the Middle East
		Formation of joint funds between Islamic countries for pharmaceuticals
	Management and development of health tourism	Determining the management of health tourism
		Reducing the power of brokers in the field of health tourism
		Receiving patients' bills in the form of international currencies
		Reducing the problems related to visas for health tourism
		Bringing needed medicines and medical devices by health tourists
		Sale of equipment at its real price (without additional costs) to health tourists
Pricing processes	Increasing expenditure on domestic medicine	Increase in the price of medicines and medical equipment together with the increase in the currency
		Addressing price instability
		Increase in the price of cheap medicines
		Continuation of the plan of branded generics and increase in the price of domestic products up to 50% of the cost of their branded counterparts
	Rationalization of the prices of medicines	Reducing import and increasing production through rational pricing of medicines
		Consideration of transportation costs in pricing
		Elimination of counterfeiting through real pricing
		Change in the pharmaceutical price according to the value of major international currencies
Management of insurance organizations	Strengthening the insurance system	Payment of the pharmaceutical subsidies to insurance organizations
		Insurance coverage to cover pharmaceutical price increase
		Complimentary insurance role for those in higher percentiles
		Crossover subsidiary
		Mandatory insurance for all people
	Removal of unnecessary medications from the list of insurance benefits	Removal of luxury and cosmetic drugs from basic coverage
		Increasing coverage for generic and low-cost brands
		Elimination of brands for which generic alternatives exist
		Revising the basic benefits package
		Revising the basic benefits package
	Transforming insurance into an industry	Research by insurance organizations on ways to reduce drug costs
		Cost-effectiveness research on pharmaceuticals
		Control of insurance companies over the way medicines are consumed
		Pricing and monitoring the funds
		Changes in insurance methods
Integrated health information system	Electronic health record	Integration of health information systems
		Use of the health care system to reduce the financial burden
		Definition of services and drugs in the integrated system
		Preparing facilities for the introduction of the smart health card
	Electronic prescription	Electronic prescribing to reduce treatment burden
		Electronic facilities in rural areas for the development of electronic prescribing
		Electronic prescribing as a tool to control prescribing
		Defining a system to select the best drugs according to the cost and symptoms of a disease
Mechanization of the distribution system	Medication tracking	Tracking of medicines from distribution to consumption
		Online relationship between MOH and distributors and importers
		Design of a mechanized system for prescribing
		Channelized distribution through a digital system
	Control and monitoring of pharmacies and distributors	Prevention of hoarding of medicines in pharmacies
		Prevention of the over-the-counter sale of medicines in pharmacies
		Reduction in the number of brokers in the distribution system
		Reduction in the number of distributors
		Correct planning for daily exclusion of medicines from distribution companies
Role of suppliers and manufacturers	Provision of high-quality medicines	Increasing the quality of domestic drugs and medical devices
		More attention to the quality of domestic supplements
		In vivo and in vitro evaluation of drugs by the government
		Evaluation of the source substance of medicines by the MOH
		Improving the quality of domestic production through interaction between manufacturers, prescribers, and consumers
		Increasing the warranty of domestic products
	Increasing production and exports	More planning to increase domestic products
		Production of medicines and medical devices for domestic use and export
		Attention to the export of high-tech drugs and generics
		Financing the provision of the initial substance through the development of drug export
		Profitability through large-scale drug production
Role of the medical community and consumers	Informing the medical community	Increasing the knowledge of the medical community about domestic products
		Increasing physicians’ and pharmacists’ knowledge about the costs and combinations of medicines
		Strengthening the relation between pharmacies and the physicians
		Physician access to the software containing drug lists, costs, and indication for prescribing
		Raising physician awareness of the national pharmacopeia
		Developing a culture of rational use of medicines and prohibiting self-medication
		Emphasizing traditional Iranian medicine
		Prohibition of prescribing non-indicated and expensive medicines
	Use of alternative medicines and methods	Use of simple and effective pharmaceutical protocols
		Not using the most sophisticated methods for all cases
		Justification and encouragement of patients to use alternative medicines
		Substitution of monopolistic drugs by others
		Determining the alternatives of 1,2,3 of treatment
		Attention to the treatment priorities and collaborative decision making
	Role of providers regarding lack of brand loyalty	Less attention to international brands
		Greater oversight over the prescription of branded drugs
		Prescribers’ lack of vested interest in recommending a certain manufacturer’s products
		Improving medical ethics to limit branding
		Providers’ and prescribers’ positive attitude towards domestic drugs
	Consumer–patient collaboration	Rational prescribing of medications
		Improving patients' culture and knowledge about medication use
		Encouraging patients to use domestic medicines

The main themes included strategies for: ‘health care management and policy’, ‘leveraging local potential’, ‘strengthening relationships with other countries’, ‘pricing’, ‘insurance organizations’, ‘health information system integration’, ‘distribution system mechanization’, ‘suppliers and manufacturers’, and ‘medical community and consumers’.

### Strategies for healthcare management and policy

Health policy and management as a strategy to increase the resilience of the pharmaceutical supply chain in the event of politico-economic sanctions included six subthemes: revising Iran's health management, expanding and strengthening supervision, providing clinical guidelines, delegation and privatization, centralized and strategic purchasing, and properly implementing the GP plan and referral system.

Implementing revisions in Iranian healthcare management to pay more attention to health economics, considering the efficiency of the healthcare system, and developing related plans and programs were highlighted by some interviewees as a strategy to increase the resilience of the pharmaceutical supply chain to sanctions. In this regard, one of the participants opined that:

“Iran's pharmaceutical and healthcare industry is threatened by many natural and human factors. Therefore, I think all countries need to have preparation and resilience plans to deal with these threats to minimize the damage" [P_4_].

Some interviewees pointed out that increasing and strengthening oversight was a strategy to increase the resilience of the pharmaceutical supply chain to sanctions. In this regard, a participant noted:

"There are oversight tools, but how effective they are questionable, and it depends to some extent on the individual and the health system. For example, universities in Iran have a very different range of oversight procedures. In my opinion, in this case, the headquarters of the Ministry of Health and Medical Education need to standardize them more seriously" [P_12_].

Many respondents highlighted the role of clinical guidelines in improving the resilience of the pharmaceutical supply chain to sanctions. In this regard, one of the respondents said the following:

"Although clinical guidelines exist in other countries to help doctors provide the right treatment, they are lacking in our country" [P_2_].

Most respondents also recognized delegation and privatization as a useful strategy to increase the resilience of the pharmaceutical supply chain to sanctions. For example, one of the participants reiterated that:

"The Ministry of Health and Medical Education must increase the powers and duties of universities and the private sector and delegate them to university circuits, private companies, and cooperatives. The IT department must also monitor the activities and make policies without interference in their practice" [P_1_].

Almost half the respondents believed that centralized and strategic purchasing could be an effective strategy to increase the pharmaceutical supply chain's resilience to sanctions. In this regard, one of the participants stated that:

"Pharmaceutical operations need an adequate infrastructure in terms of software, human resources, control and monitoring capabilities, and a strong financial structure, such as separate financial accounts" [P_10_].

In relation to the GP plan and referral system, one of the participants added that:

"If the GP plan is properly implemented, it can be effective; but in the current situation, it just increases medication consumption" [P_7_].

### Strategies for exploiting local potentials

This theme constituted three subthemes: increasing inter-sectoral cooperation, paying more attention to mass media, and leveraging national and religious power and authority.

Several respondents believed that increased inter-sectoral collaboration between the health industry and other sectors could help address sanctions-related issues and make the supply chain more resilient. In this regard, one of the participants said that:

"The authorities must draw up a list of shortcomings and call on universities, the private sector, and, above all, the military to produce medical items and equipment, since they have access to more sophisticated technologies" [P_1_].

Most of the participants emphasized the role of mass media in improving sanctions-related conditions and creating peace in society. For example, one of the participants affirmed:

"Mass media should report the facts; but unfortunately, managers lack good advisors to solve such problems and increase transparency in society" [P_7_].

Several participants also indicated that such challenges in the pharmaceutical supply chain could be overcome by relying on national and religious power and authority. In this regard, one of the participants stated that:

"Since we are believers, it is expected that we can solve many problems by relying on our religious and national enthusiasm" [P_13_].

### Strategies for strengthening relationships with other countries

As a strategy to improve pharmaceutical supply chain resilience, relationships with other countries constituted two subthemes: interactions with neighboring countries and health tourism development. Regarding interactions with neighboring countries, one participant said:

"Alliance and cooperation between Islamic countries and other nations and the establishment of a common monetary fund can lead to self-reliance and reduce dependence" [P_9_].

In addition, regarding the development of health tourism, one of the participants opined that:

"The real value of health services should be taken into account, and further arrangements need to be made with source countries to allow tourists to bring their medicines and equipment that they need" [P_8_].

### Pricing strategies

The process of pharmaceutical pricing as a strategy to improve the resilience of the pharmaceutical supply chain included two subthemes: increasing spending on domestic medicines and rationalizing medicine prices.

Most respondents believed that spending on domestic medicines was low and that prices should be increased. In this regard, one of the respondents added that:

"At this point, the government provides managed foreign exchange for imported commodities, but free-market agents finance other production costs. Therefore, it is expected that the government will then set drug prices based on actual costs rather than on managed foreign exchange [P_3_].

All the respondents were sure that rationalization of drug prices is a good strategy. For example, one of the participants said that:

"If the government acts logically in rationalizing drug prices and makes it much more real, manufacturing companies will spontaneously shift to production and reduce the need for imports" [P2].

### Strategies for insurance organizations

Three subthemes, namely strengthening the insurance system, removing unnecessary drugs from the list of insurance benefits, and transforming insurance into an industry, were coded for this item.

Accordingly, most respondents acknowledged that increasing insurance coverage and strengthening the insurance system could effectively increase the resilience of the pharmaceutical supply chain. In this regard, one of the respondents added that:

"Currently, not all people benefit from insurance coverage. I think insurance should become mandatory for all. Also, supplementary insurance is passive and needs to cover excess layers" [P14].

Some interviewees mentioned the need to replace drugs with cheaper alternatives as an effective strategy to improve the resilience of the pharmaceutical supply chain. For example, one participant stated that:

"The authorities should remove expensive drugs from the insurance system and increase insurance coverage for commonly used drugs" [P_2_].

A few respondents also believed it was necessary to transform insurance into an industry and conduct research activities and cost-effectiveness studies. For example, one of the respondents believed that:

"In our country, insurance does not function as an industry, and it is just like a fund to keep the money. Unfortunately, in terms of the aging population, insurance organizations are going bankrupt, and we need to use tried and tested methods to get to the bottom of this problem" [P_10_].

### Strategies for integrating the health information system

The integrated health information system included two subthemes: electronic health records and electronic prescribing.

Regarding the electronic health record, one of the participants said that:

"Currently, our information is not sufficient and integrated, so the more access we have to patient information, the more effectively we can deliver treatments. It is also of the utmost importance to create electronic health records and link them to governmental and private insurance organizations" [P_9_].

Regarding electronic prescribing, one of the participants added that:

"Proper implementation of electronic health records can actually reduce the treatment burden in Iran. However, infrastructures such as the high-speed Internet in rural areas or alternative plans and programs in the event of power outages should be considered" [P2].

### Strategies for mechanizing the distribution system

Tracking medicines and monitoring pharmacies and distributors were analyzed as two subthemes that can make the pharmaceutical supply chain more resilient.

Specifically, all respondents emphasized the mechanization of the distribution system and believed that pharmaceutical products need to be tracked and controlled from the moment they are manufactured or imported into the country. In this regard, one of the respondents said that:

"The national distribution center must be equipped with a tracking system for the distribution of drugs; this will solve many of the problems through the mechanization of the integrated distribution system and determining the exact number of each drug in every corner of the country" [P_8_].

Regarding the supervision of pharmacies and distributors, one of the participants said that:

"Besides increasing the efficiency of the supply chain, reducing the number of distributors can also significantly affect the level of control over them" [P4].

### Strategies regarding the role of suppliers and manufacturers

This main theme included two subthemes, namely providing quality medicines and increasing production and exports.

In relation to high-quality medicines, one of the participants said that:

"The Ministry of Health and Medical Education must evaluate in vivo and in vitro raw materials. However, most of the assessments are quantitative in nature and only check whether the drug dose is given or not, but the effects of the drugs on the patient's body are not assessed" [P5].

Regarding increasing production and export, one of the participants also affirmed:

"Fortunately, there is good capacity and potential in the country, and we are equipped with human capital and intellectual resources to take advantage of this opportunity to increase production and export, especially in the area of high-tech drugs" [P_2_].

### Strategies regarding the role of the medical community and consumers

This main theme included four subthemes: medical community information, alternative medicines and methods, brand loyalty, and consumer–patient collaboration.

Accordingly, most respondents believed that the medical community should be informed of the prescription of alternatives. In this regard, one of the participants said that:

"There are quite a number of alternatives to imported drugs and supplements in the country, but prescribers do not know about their differences and ingredients. Given their exorbitant prices, prescribing foreign drugs can be thoughtless and insensitive. I think we need to provide more information to the medical community in this regard" [P1].

Regarding the use of alternative medicines and methods, one participant added:

"We need to move toward using alternative means for treatments. We also need to have alternative protocols" [P_8_].

Regarding the role of providers in the lack of brand loyalty, one of the participants said that:

"It seems that physicians' loyalty to a brand should be based on scientific evidence as well as the quality of medicines, but brand orientation is based solely on advertising and marketing by pharmaceutical representatives" [P_10_].

All the respondents similarly believed in the role of the patient and consumer in rationalizing the use of medicines. In this regard, one of the participants added that:

"The flow of movement should be towards the rational use of medicines by patients. So I think we need to build and promote a culture of rational use of medicines" [P3].

**Fig. 1:**
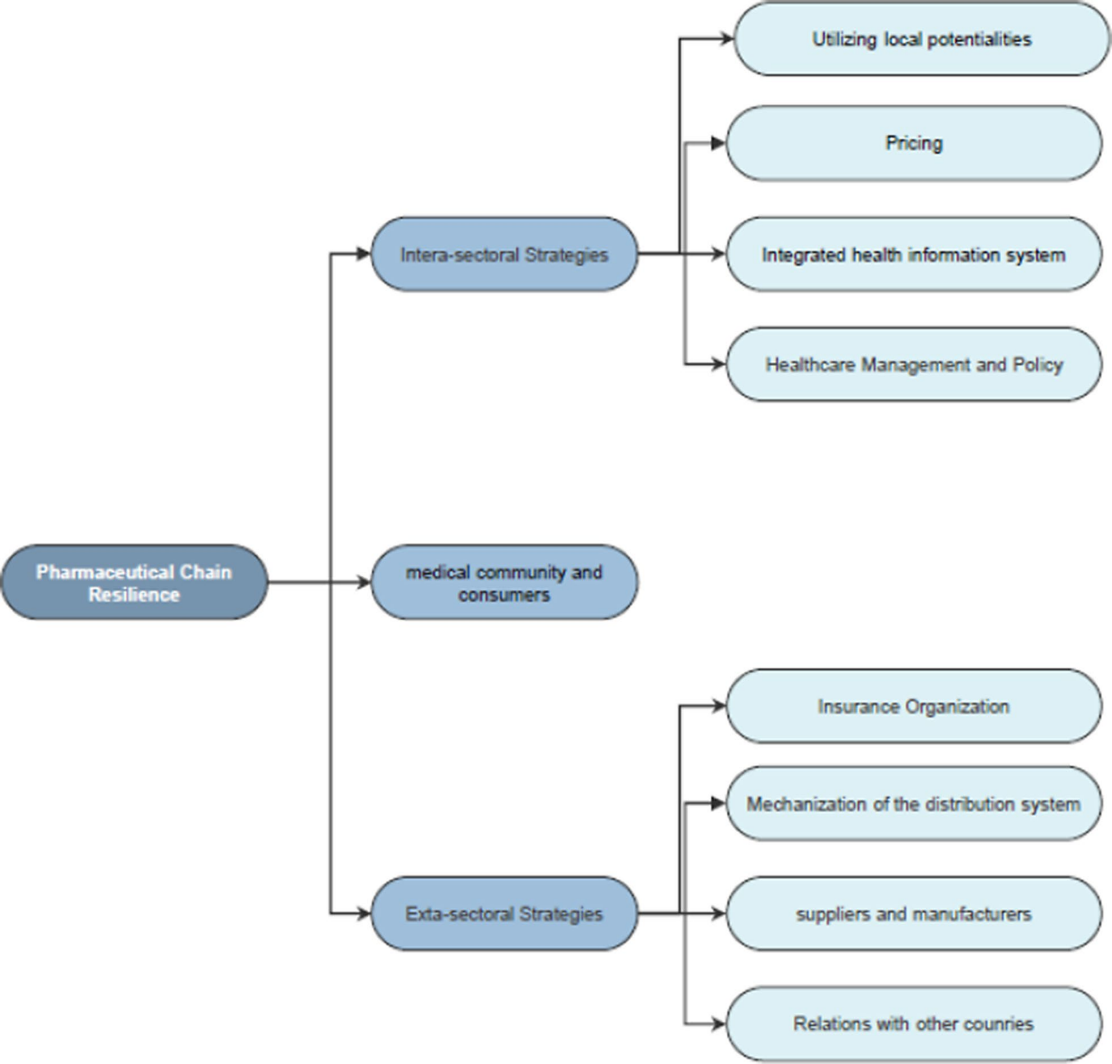
Thematic map of the study

A thematic map was created to better illustrate the information and highlight the most representative themes (Fig. 1). According to Fig. 1, some of these strategies have an extra-sectoral character, such as 'insurance organizations', 'strengthening relations with other countries', 'mechanization of the distribution system', and 'suppliers and manufacturers.' At the same time, some inter-sectoral strategies can help the pharmaceutical chain to maintain its resilience. These strategies include 'health management and policy', 'exploiting local potential', 'pricing', and 'integrated health information system'. As a strategy, 'Medical community and consumers' also plays an important role in this area.

## Discussion

The findings of this study revealed strategies to improve resilience and address the challenges of the pharmaceutical supply chain under politico-economic sanctions. These strategies would enable the pharmaceutical supply chain to be more resilient at both national and industrial levels. This fundamentally helps maintain pharmaceutical services' availability at a high level, leading to a satisfied community. Indeed, resilience offers the pharmaceutical supply chain a greater opportunity to reconfigure itself as the environment changes.

As mentioned earlier, healthcare management and policy would be a powerful tool to respond to environmental changes. More specifically, the negative effects of sanctions can be mitigated by adopting appropriate policies. Cuba is a good case in point. Adopting appropriate pharmaceutical supply chain policies has managed to mitigate the negative effects of sanctions over time [18]. It goes without saying that health policies need to be reformed for a health system to find the best way to respond to market needs [19]. Such a policy illuminates all activities with a greater focus on drug policy to ensure that society can meet its drug needs. More specifically, reliance on local potentials, if well formulated, would provide the country with a high level of resilience in the pharmaceutical supply chain. This is essentially through increased added value in the supply chain with less cost and more productivity. Such an approach has been shown to significantly minimize the risk of drug scarcity and access in the pharmaceutical supply chain over the last decade [20]. More importantly, attention to local potentials creates a virtuous cycle that helps build more capabilities over time to cope with imposed constraints [21]. This view is entirely applicable in a country with a large population and a highly recognized human capital.

It should be noted that investing in local capacity does not rule out the potential in neighboring countries; rather, this capacity is a very cost-effective tool to ensure resilience in a supply chain [22]. Therefore, as participants emphasized, policies must be formulated to benefit from both local and neighboring capacity. This is particularly important in the case of Iran, as the country is located in the Middle East region and has good access to the fourth corner of the world [23].

As for the pricing strategy, the World Health Organization (WHO) has proposed it as an effective tool to address the challenges of sanctions against medicines by rationalizing prices [24]. This contributes significantly to the rational use of medicines, leading to a reduction in wastage of medicines, especially those subsidized by the government. For example, sensible pricing can potentially discourage self-medication. The money saved through this policy would be spent on other prescribed drugs to protect the population from high costs. This policy is well supported if insurance companies remove unnecessary drugs from the positive list and exclude the expensive drugs that do not clinically add value to the patients. To this end, such companies should apply an appropriate rationing system to achieve clinical efficiency, safety, and access [25]. More importantly, an integrated health information system wonderfully enables the healthcare system to provide the right medicines to the right people [26]. This is mainly done by providing electronic health records and electronic prescriptions. Finally, the medical profession and consumers play a critical role in providing resilience in the pharmaceutical supply chain. Physicians can change prescribing behavior and provide alternative medications that are cost-effective and reduce patient co-payments. Physicians are key players in the healthcare system as they are in the right position to convince patients to follow the recommended path. On the other hand, consumers would be well informed about their situation and the alternatives offered to them. Building mutual trust is a very powerful tool in ensuring respect and de-escalating conflicts.

### Limitations

This study has some limitations. First, it is better to triangulate the qualitative data obtained through the interviews with other types of possible data, such as the national documents and reports. Second, the views of the community, producers, and traders may enrich the results, which should be considered in further studies.

## Conclusions

According to the present study, some strategies were found to make the pharmaceutical supply chain more resilient under politico-economic sanctions. In this regard, policymakers need to consider the integration of pharmaceutical supply chain activities with modern technologies, especially in the case of large-scale pharmaceutical industries, attention to business complexity, economic development, intense competition, rapid changes in customer needs, and appropriate relationship between manufacturers, distributors, prescribers, and insurance organizations as buyers.

## Data Availability

They are available upon reasonable request to the corresponding author.
